# Myopia: a growing global problem with sight-threatening complications

**Published:** 2015

**Authors:** Brien A Holden, David A Wilson, Monica Jong, Padmaja Sankaridurg, Timothy R Fricke, Earl L Smith III, Serge Resnikoff

Globally, myopia is the leading cause of distance refractive error, affecting 1.45 billion or 27% of the world's population in 2010 (myopia being defined as more than or equal to 0.50 D of myopia).^1^ The number of people with myopia is expected to continue to rise both in absolute numbers and as a percentage of the population.^2^ In certain age groups in several Asian countries, the prevalence of myopia is over 80%. Among late teenagers and young adults in Korea, Taiwan and China the prevalence is now between 84% and 97%.^3–5^

In addition to the increase in prevalence, there is evidence of a rise in the severity of myopia.^3^ A study by Vitale *et al.*^6^ in the United States found that the prevalence of moderate myopia, defined as between −2.00 D and −7.9 D, nearly doubled (from 11.4% in 1971–1972 to 22.4% in 1999–2004), and that the prevalence of high myopia, which was defined as more than 8.00 D of myopia for this study, had increased eightfold during the same period (from 0.2% to 1.6%). The global prevalence of high myopia (commonly defined as greater than or equal to 5.00 D of myopia) was 2.9% (224 million people) in 2010.^7^

## Risks

High myopia is associated with an increased risk of developing sight-threatening conditions such as myopic macular degeneration (defined as atrophic changes or choroidal neovscularisation in the macular region in high myopia), retinoschisis, posterior staphyloma, glaucoma retinal detachment, and cataract.^8,9^ A literature review found that the prevalence of vision impairment due to pathologic myopia (high myopia with one or more typical fundus lesions) is between 0.1% and 0.5% in European studies and between 0.2% and 1.4% in Asian studies.^9^ In a Japanese study, 12.2% of vision impairment was caused by pathologic myopia.^10^ Myopic macular degeneration has been reported to be the major cause of monocular blindness in Tajimi, Japan,^11^ and the leading cause of new cases of blindness in Shanghai, China.^12^ Without interventions to slow the progress of myopia, the prevalence of pathologic myopia can be expected to increase.

**Figure:**
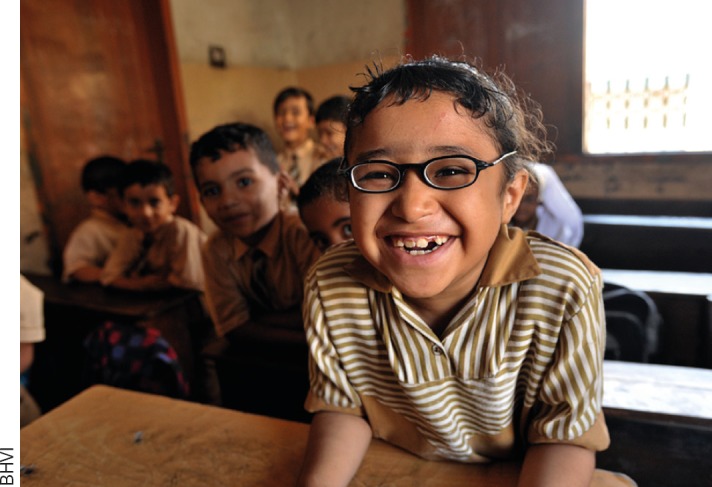
A child with myopia. PAKISTAN

## Causes and cures

Myopia development and progression is considered to be multifactorial, with genetic and environmental factors contributing, although the contribution of genetics is considered small. Genes have been identified for myopia^13,14^ but genes are thought to determine one's susceptibility to environmental factors.^15^ Several environmental factors have been identified,^15–18^ including too much time spent on near work;^18^ insufficient time spent outdoors;^19^ low levels of vitamin D;^20^ inadequate light exposure;^21^ and poor diet.^22^ There is evidence emerging that increased time spent outdoors can reduce the risk of developing myopia and – in those with myopia – it can reduce the rate of progression.^23,24^ Reduced rates of progression in summer compared to winter^25^ also supports the hypothesis of the light theory. It has also been shown in experimental animal models that a defocused retinal image can lead to axial elongation of the eye, and hence myopia. While it seems unlikely that there will be a treatment that can completely prevent myopia development and progression, there are some promising treatments on the horizon. These include providing a focussed image at all retinal locations to remove the stimulus to axial eye elongation. Executive bifocal spectacles,^26^ peripheral plus contact lenses,^27^ extended depth of focus contact lenses^28^ and orthokeratology^29^ are some of the optical intervention methods that show some efficacy in reducing the rate of progression of myopia (some by up to 51%). Pharmacological agents such as 0.01% atropine are being prescribed in Asia, and show a reduction of up to 50% in the rate of progression of myopia, although there is no reduction in the rate of axial elongation.^30^ Oral tablets containing 7-methylxanthine (7-MX) have been approved for use in children in Denmark for myopia control and shows some efficacy, but long-term studies are needed.^31^ While these approaches have been tried in isolation, they may have a greater impact if different approaches are combined.

## Discussion

Uncorrected distance refractive error is already a major global health problem. It is the main cause of vision impairment and the second highest cause of blindness.^32^ It has also been estimated to cost about US $202 billion^33^ in global lost productivity. However, whereas uncorrected refractive error is the second major cause of blindness, this does not include blindness from pathologic myopia; nor does the estimate of the financial burden of uncorrected refractive error take into account blindness due to pathologic myopia.

Myopia can no longer be considered a benign refractive error, easily corrected with a pair of spectacles or contact lenses. It has an insidious side which, even if adequately corrected, can sometimes progress and may lead to sight-threatening complications. Current forms of correction for myopia (spectacles, contact lenses and refractive surgery), do not cure the underlying myopia but provide an optical solution for clear vision and thus do not offer any protection from the consequences of high myopia.

Given that myopia is already one of the major causes of vision impairment and blindness, and is projected to affect almost half of the world's population within 40 years,^2^ urgent action is demanded from governments, non-government organisations and researchers. Policy makers must recognise the risk of increasing myopia and ensure that appropriate detection and treatment is available. Myopia control is possible but clinicians must adopt myopia control strategies as soon as a child becomes myopic. Parents should also be encouraged to monitor the time their children spend on near devices and encourage time spent outdoors.

Visit www.cehjournal.org to view all the references associated with this article. **Further reading:** www.mivision.com.au/high-myopia-prevalence/

